# Does intravascular ultrasound provide clinical benefits for percutaneous coronary intervention with bare-metal stent implantation? A meta-analysis of randomized controlled trials

**DOI:** 10.1186/2046-4053-1-42

**Published:** 2012-09-21

**Authors:** Lucas Lodi-Junqueira, Marcos Roberto de Sousa, Leonardo Carvalho da Paixão, Silvana Márcia Bruschi Kelles, Carlos Faria Santos Amaral, Antonio L Ribeiro

**Affiliations:** 1 Instituto de Avaliação de Tecnologias em Saúde (IATS), do Hospital das Clínicas da Universidade Federal de Minas Gerais (UFMG), Avenida Alfredo Balena, 110, CEP, 30130-100, Belo Horizonte, MG, Brazil; 2 Setor de Hemodinâmica do Hospital das Clínicas da UFMG, Avenida Alfredo Balena, 110, CEP, 30130-100, Belo Horizonte, MG, Brazil; 3 Departamento de Clínica Médica da Faculdade de Medicina da UFMG, Avenida Alfredo Balena, 190, CEP, 30130-100, Belo Horizonte, MG, Brazil

**Keywords:** Intravascular ultrasound, Meta-analysis, Publication bias, Bare-metal stent, Percutaneous coronary intervention, Coronary artery disease

## Abstract

**Background:**

The role of intravascular ultrasound (IVUS) in percutaneous coronary interventions (PCI) is still controversial despite several previously published meta-analyses. A meta-analysis to evaluate the controversial role of IVUS-guided PCI with bare-metal stenting was performed and a previous published meta-analysis was re-evaluated in order to clarify the discrepancy between results of these studies.

**Methods:**

A systematic review was performed by an electronic search of the PubMed, Embase and Web of Knowledge databases and by a manual search of reference lists for randomized controlled trials published until April 2011, with clinical outcomes and, at least, six months of clinical follow-up. A meta-analysis based on the intention to treat was performed with the selected studies.

**Results:**

Five studies and 1,754 patients were included. There were no differences in death (OR = 1.86; 95% CI = 0.88-3.95; p = 0.10), non-fatal myocardial infarction (OR = 0.65; 95% CI = 0.27-1.58; p = 0.35) and major adverse cardiac events (OR = 0.74; 95% CI = 0.49-1.13; p = 0.16). An analysis of the previous published meta-analysis strongly suggested the presence of publication bias.

**Conclusions:**

There is no evidence to recommend routine IVUS-guided PCI with bare-metal stent implantation. This may be explained by the paucity and heterogeneity of the studies published so far.

## Background

Since the first studies of intravascular ultrasound (IVUS) were published in 1989 [1-4], the technique has been widely used in clinical research and has contributed to technological improvements in interventional cardiology [5]. As a diagnostic tool, IVUS helps in the assessment of coronary lesions classified as moderate based on angiography, especially those located in the left main coronary artery [6,7], and in the assessment of long lesions, small artery lesions, bifurcations and in-stent restenosis [8,9]. As an ancillary technique in percutaneous coronary intervention (PCI), IVUS is useful in the evaluation of the target lesion and during stent implantation [10]. In theory, its use should reduce the risk of major adverse cardiovascular events (MACE) because of lower restenosis and stent thrombosis rates.

The first published systematic review evaluated the role of IVUS in PCI as well as its cost-effectiveness and did not show any difference between IVUS and angio-guided PCI [11]. A few years later, a meta-analysis did not show any reduction in death or myocardial infarction (MI) but revealed reductions in repeat revascularization and angiographic restenosis after a six-month follow-up [12]. This was corroborated by another meta-analysis that suggested an improvement in acute post-interventional results (larger minimal luminal diameter) and lower repeat revascularization, angiographic restenosis and MACE rates, but showed no effect on death or MI during the follow-up period of six to thirty months [13].

Since IVUS clinical benefit is still controversial and conclusions of meta-analyses may be misleading due to methodological issues, we performed a meta-analysis to assess the effect of IVUS in PCI with bare-metal stent implantation on clinically relevant outcomes, assessing the presence of publication bias. In addition, a critical review of the last published meta-analysis [13] was performed in order to clarify the discrepancy in the results found in this analysis comparing to medical literature.

## Methods

The protocol for the present systematic review was based on the PRISMA Statement [14] and it was registered in the PROSPERO database (CRD42012002767).

### Strategy search

We performed an electronic search of PubMed, Embase and Web of Knowledge databases with the following terms: Myocardial Ischemia; Ischemic Heart Disease; Acute Coronary Syndrome; Angina; Coronary Disease; Coronary Artery Disease; Coronary Occlusion; Coronary Thrombosis and Myocardial Infarction, in association with the terms Interventional Ultraso*; Intravascular Ultraso*; Intracoronary Ultraso*; IVUS and ICUS.

A manual search was also performed to retrieve potential articles cited in previous meta-analyses, in review articles and those considered to be relevant by the reviewers. The electronic search, which evaluated the articles included in the databases through April 2011, was limited neither by publication date nor by language.

### Eligibility criteria

Only randomized controlled trials that compared IVUS-guided PCI with angiography-guided PCI, with clinical outcomes, and at least six months of clinical follow-up, were included in quantitative synthesis. The clinical outcomes considered were death, nonfatal MI and the combined endpoint of MACE (death, nonfatal MI, or repeat revascularization). For repeat revascularization, a report of any new coronary revascularization (surgical or percutaneous) was considered, regardless of the lesion and of the vessel treated. Surrogate outcomes, such as angiographic outcomes, were not taken into account because these can show a positive result with no effect (or harmful effect) on clinical outcomes [15]. These clinical outcomes (death, nonfatal MI and MACE) were considered primary endpoints in our meta-analysis.

### Study selection

The titles and abstracts from the articles retrieved by the search strategy had been independently evaluated by two reviewers (LCP, LLJ). All articles in which IVUS was mentioned were selected. These articles were fully read, and those that met the criteria were included. Disagreements were solved by consensus. If consensus was not achieved, a third reviewer (ALR) defined the question.

### Statistical analysis

The intention-to-treat meta-analysis that followed the systematic review was performed by the random-effects model of the Comprehensive Meta-Analysis software (Borenstein M, Hedges L, Higgins J, Rothstein H. Version 2.2.048, Biostat, Englewood NJ, USA 2005), with the odds ratio (OR), 95% confidence intervals and two-sided *P*-values calculated for each outcome. The analysis of heterogeneity between studies was estimated by the *I*^*2*^ statistic.

Publication bias evaluation was performed by Duval and Tweedie’s Trim and Fill method [16]. Egger's test was also performed to analyze the impact of several factors on the size of the treatment effect [17]. The small study effect was also evaluated by cumulative analysis (from largest to smallest sample size) and by the one-study-removed method.

## Results

### Literature search

**Figure 1:**
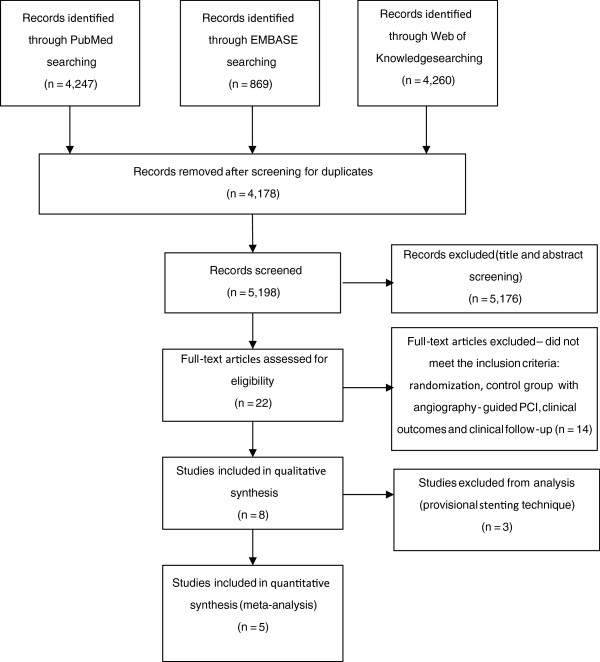
**Article selection flowchart.** Flowchart based on the PRISMA Flow Diagram [14].

A total of 4,247 articles in PubMed, 869 in Embase and 4,260 in Web of Knowledge databases were identified. Eight studies were selected according to the inclusion criteria (Figure 1) [18-26]. After a comprehensive analysis, three studies were excluded because they used a provisional stenting technique [19,25,26], which is no longer performed because of its higher restenosis rate [27]. Table 1 summarizes the clinical and angiographic characteristics of the patients included in the selected studies.

**Table 1:** Patient characteristics

**Study**	**DIPOL**		**AVID**		**RESIST**		**TULIP**		**OPTICUS**	
	**IVUS**	**QCA**	**IVUS**	**QCA**	**IVUS**	**QCA**	**IVUS**	**QCA**	**IVUS**	**QCA**
Demographic characteristics										
Patients, n	83	80	369	375	79	76	73	71	273	275
Age. years, mean ± SD	56 ± 8	54 ± 8	62 ± 12	63 ± 11	57 ± 10	56 ± 12	63 ± 10	61 ± 10	60.1 ± 10	61.5 ± 9.5
Men,%	71	73	73	68	86	93	71	72	77	78
Smoker,%	47	52	-	-	55	51	40	43	0.69	0.66
Previous MI,%	44	40	35	29	54	48	-	-	0.32	0.32
Previous CABG,%	-	-	18	20	-	-	-	-	0.03	0.04
Previous PCI,%	-	-	24	25	-	-	-	-	0.2	0.2
Diabetes mellitus.%	10	11	15	17	9	8	21	16	0.17	0.17
Dyslipidemia,%	47	40	40	44	54	52	62	61	0.61	0.67
Hypertension,%	-	-	46	45	24	26	30	27	0.48	0.52
LV ejection fraction,%, mean ± SD	52 ± 9	48 ± 10	53 ± 13	55 ± 13	51 ± 9	53 ± 13	-	-	56.5 ± 14	57.7 ± 14.3
Angiographic characteristics										
Target vessel,%										
- Left anterior descending artery	41	46	40	37	48	47	39	38	51	50
- Left circumflex artery	26	24	15	18	11	11	10	21	18	14
- Right coronary artery	33	30	35	32	41	42	51	41	30	35
- Left main coronary artery	-	-	0.8	0.5	-	-	-	-	-	-
Lesion length, mm, mean ± SD	N/A	N/A	13.0 ± 7.7	13.3 ± 9.2	7.7 ± 3.5	8.0 ± 4.0	27.0 ± 9	29.0 ± 10	11.9 ± 5.1	11.6 ± 5.5
Reference diameter, mm, mean ± SD	3.21 ± 0.64	3.19 ± 0.59	3.05 ± 0.64	3.00 ± 0.54	3.0 ± 0.64*	2.89 ± 0.54*	2.95 ± 0.57	2.96 ± 0.53	2.97 ± 0.53	3.01 ± 0.51
Type B2 or C ACC/AHA lesions,%	13	10	N/A	N/A	43	48	100	100	76	78
Pre-intervention										
- Minimum lumen diameter, mm, mean ± SD	0.97 ± 0.33	0.95 ± 0.32	1.11 ± 0.5	1.09 ± 0.47	0.96 ± 0.37	1.02 ± 0.44	1.02 ± 0.42	0.99 ± 0.41	0.96 ± 0.35	0.99 ± 0.34
- Diameter stenosis,%, mean ± SD	69.7 ± 14.2	70.2 ± 11.4	63.4 ± 14.1	63.5 ± 14.3	65.0 ± 11.0	64.0 ± 12.0	65.0 ± 13.0	65.0 ± 10.0	67.6 ± 11.2	66.7 ± 10.1
Post-intervention										
- Minimum lumen diameter, mm, mean ± SD	3.34 ± 0.55	3.06 ± 0.52	2.93 ± 0.55	2.87 ± 0.48	2.48 ± 0.43	2.46 ± 0.46	3.01 ± 0.40	2.80 ± 0.31	3.02 ± 0.49	2.91 ± 0.41
- Diameter stenosis,%, mean ± SD	3.4 ± 2.9	8.9 ± 5.4	N/A	N/A	19.0 ± 10.0	19.0 ± 9.0	12.0 ± 7.0	13.0 ± 9.0	2.8 ± 7.8	6.0 ± 8.0

* In the RESIST study, the reference diameter average was calculated. MI, myocardial infarction; CABG, coronary artery bypass graft; PCI, percutaneous coronary intervention; LV, left ventricular; ACA, American College of Cardiology; AHA, American Heart Association; IVUS, intravascular ultrasound; N/A, not applicable, QCA, Quantitative Coronary Angiography.

### Qualitative study analysis

There were significant differences between the five studies included in the final analysis (Table 2). One of the current indications of IVUS-guided PCI is for patients with long lesions (greater than 15 or 25 mm) [8,28,29], who have been excluded from most studies [18,20,24]. Unlike the others, the TULIP study excluded those patients who had focal lesions (less than 20 mm in length). Every study but the AVID trial excluded patients with a current or past history of acute coronary syndrome (ACS). In the RESIST study, randomization was performed only after the intervention, which may have caused a selection bias. In the AVID trial, the IVUS analysis was only performed after implantation of the stent, excluding the initial assessment of the target lesion [8].

**Table 2:** Study characteristics

**Study**	**DIPOL**	**AVID**	**RESIST**	**TULIP**	**OPTICUS**
Enrolling years	2000 to 2002	1995 to 1998	1995 to 1997	1991 to 2001	1996 to 1998
Randomized	Yes	Yes	Yes	Yes	Yes
- Blinded*?*	Yes	Yes	N/A	N/A	Yes
*-* When?	Pre-intervention	Pre-intervention	Post-intervention	Pre-intervention	Pre-intervention
Intention-to-treat analysis	No	Yes	No	Yes	Yes
Exclusion criteria					
*-* Long lesions	Yes (> 25 mm)	No	Yes (> 15 mm)	No	Yes (> 25 mm)
*-* Bifurcation	Yes	No	No	Yes	Yes
*-* Left main coronary artery	Yes	Yes	No	No	Yes
*-* Chronic total occlusion	Yes	Yes	Yes	Yes	No
*-* Recent acute coronary syndrome	Yes	No (except MI with TIMI flow grade < 3)	Yes	Yes	Yes
- Small vessels	Yes (≤ 2.75 mm)	Yes (< 2.5 mm)	Yes (< 3 mm)	Yes (< 3 mm)	Yes (< 2.5 mm)
- Others	Age < 18 and > 70 y; extensive calcification; saphenous vein graft lesions	Age < 18 y; non-covered dissection; large vessels (> 3.25 mm)	Previous CABG	Focal (< 20 mm) or ostial lesions	
Pre-intervention IVUS	Yes	No	No	Yes	Yes
Post-intervention IVUS	Yes	Yes	Yes	Yes	Yes
PCI success criteria	Stent CSA/average CSA > 80%, complete apposition, stent CSA > 7.5 mm^2^	Stent CSA/distal CSA > 90%, complete apposition, no dissection	Stent CSA/average CSA > 80%	Stent MLD/average MLD > 80%, complete apposition, stent CSA ≥ distal CSA	Stenosis < 10% and MUSIC study criteria [10]
*-* PCI success by IVUS,%	96	63	61	89	82.2 and 56 (MUSIC)
Clinical follow-up, months	6	12	18	12	12
Angiographic follow-up, months	6^*^		6 (com USIC)	6	6
MACE	Death, nonfatal MI, repeat revascularization^†^	Without explicit criteria	Death, repeat revascularization^†^	Death, nonfatal MI, clinical TLR	Death, nonfatal MI, repeat revascularization^†^

^*^Angiographic follow-up was left to the discretion of the operator: 87.9% (IVUS-guided group) and 83.7% (angio-guided group). ^†^CABG or repeat PCI for any reason. IVUS, interventional ultrasound; PCI, percutaneous coronary intervention; MACE, major adverse cardiovascular events; CSA, cross sectional area; MI, myocardial infarction; TIMI, thrombolysis in myocardial infarction; CABG, coronary artery bypass graft; MLD, minimum lumen diameter; clinical TLR, ischemia-driven target lesion revascularization.

The criteria for optimal stent implantation were heterogeneous. Only the OPTICUS study used the criteria proposed by the MUSIC study [10]. The majority of patients underwent angiographic assessment after six months (angiographic follow-up) [20,22,24]. Another difference between the studies was in the criteria used for MACE. In the RESIST study, MI was not included. In the TULIP study, the MACE criteria included death, nonfatal MI and ischemia-driven target lesion revascularization (TLR). In the AVID trial, the composition of this outcome was not explained. In the other studies, the criteria for repeat revascularization were more comprehensive and included coronary artery bypass grafting (CABG) or a repeated PCI for any reason [18,20,21,24].

### Heterogeneity

The heterogeneity among the studies showed intermediate values in nonfatal MI (*I*^*2*^ = 48.82%) and MACE (*I*^*2*^ = 57.38%). For death, no heterogeneity was observed among the studies (*I*^*2*^ = 0%).

### Publication bias

**Figure 2:**
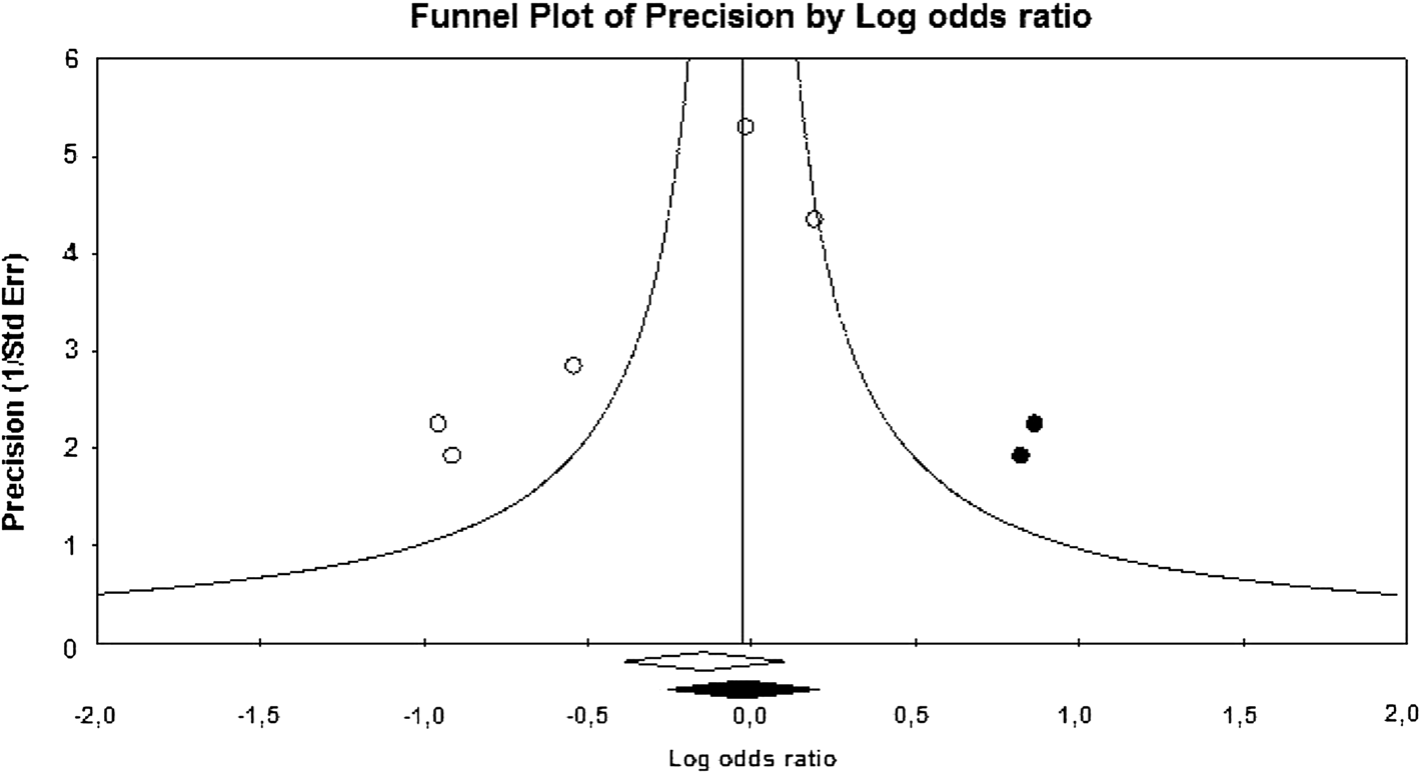
**Duval and Tweedie’s trim and fill test.** The funnel plot shows the observed studies (white circles) and the imputed studies (black circles) in addition to the observed (white diamond) and adjusted combined effect (black diamond).

We also evaluated the possibility of publication bias (B0) for MACE. Egger’s Test (B0 = −3.43; 95% CI − 6.40 to −0.47, one-tailed *P-*value 0.02) and the trim and fill test (observed OR 0.74, 95% CI 0.49 to 1.13; two studies imputed: adjusted OR 0.93, 95% CI 0.60 to 1.44) (Figure 2) were positive, suggesting the presence of small studies effects, which can be attributable to differences in design (not detected) or to publication bias.

### Meta-analysis results

**Figure 3:**
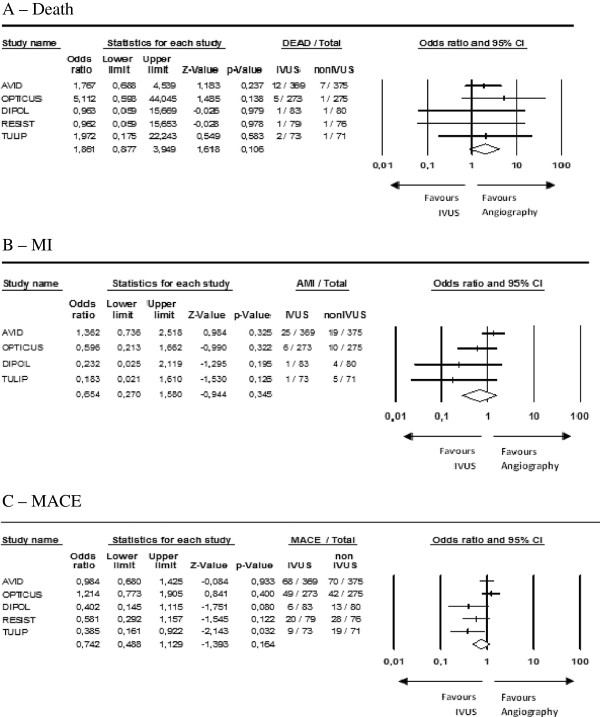
**Meta-analysis by outcomes (random effects).** (**A**) Death. (**B**) Myocardial infarction (MI). (**C**) Major adverse cardiovascular events (MACE).

A total of 1,754 patients were randomized in five studies. There was no statistically significant difference between the IVUS-guided group and the angiography-guided group (Table 3) for death (OR 1.86, 95% CI 0.88 to 3.95, *P* = 0.10) (Figure 3-A), nonfatal MI (OR 0.65, 95% CI 0.27 to 1.58, *P* = 0.35) (Figure 3-B) or MACE (OR 0.74, 95% CI 0.49 to 1.13, *P* = 0.16) (Figure 3-C).

**Table 3:** Clinical Outcomes

**Study**	**DIPOL**		**AVID**		**RESIST**		**TULIP**		**OPTICUS**		**Total**	
	**IVUS**	**QCA**	**IVUS**	**QCA**	**IVUS**	**QCA**	**IVUS**	**QCA**	**IVUS**	**QCA**	**IVUS**	**QCA**
Patients, n	83	80	369	375	79	76	73	71	273	275	877	877
Death, n (%)	1 (1.2)	1 (1.3)	12 (3.3)	7 (1.9)	1 (1.3)	1 (1.3)	2 (2.7)	1 (1.4)	5 (1.8)	1 (0.36)	21 (2.4)	11 (1.3)
Nonfatal MI, n (%)	1 (1.2)	4 (5)	25 (6.8)	19 (5.1)	N/A	N/A	1 (1.4)	5 (7.0)	6 (2.2)	10 (3.6)	33 (4.1) ^1^	38 (4.7) ^1^
MACE, n (%)	6 (7.2)	13 (16.2)	68 (18.4)	70 (18.7)	20 (25.3)	28 (36.8)	9 (12.3)	19 (26.8)	49 (17.9)	42 (15.3)	152 (17.3)	172 (19.6)

^*^The analysis of nonfatal MI excludes the RESIST Study, where this outcome was not calculated. MI, myocardial infarction; MACE, major adverse cardiovascular events; IVUS, interventional ultrasound.

### Reviewing published data

**Figure 4:**
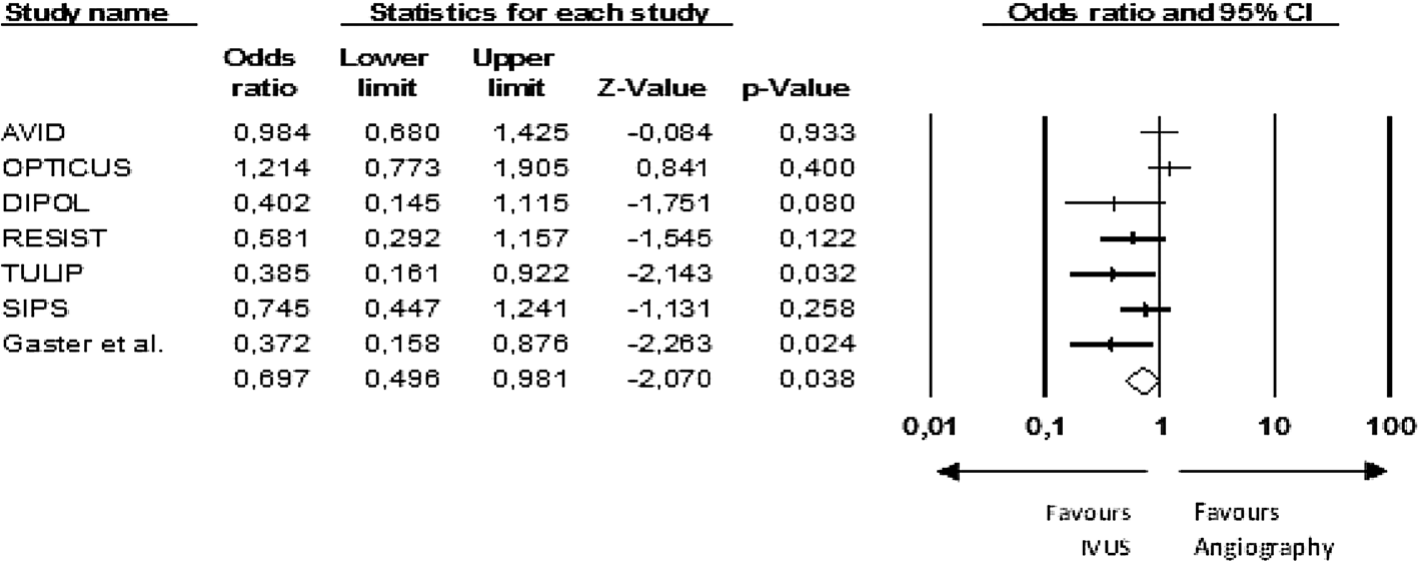
**Original data from the re-evaluated meta-analysis - MACE**[13]**.**

In order to clarify the discrepancy in MACE results found in this analysis compared to the medical literature, the data of a previously published meta-analysis [13] were re-evaluated (Figure 4). Among the studies selected by that meta-analysis, only two were not included in the present selection because the provisional stenting technique was employed in both of them [19,25].

A funnel plot analysis was performed along with Egger’s Test (B0 = −3.66, 95% CI − 5.54 to −1.78, one-tailed *P*-value = 0.002) and the trim and fill test (observed OR 0.70, 95% CI 0.50 to 0.98; three studies imputed: adjusted OR 0.89, 95% CI 0.62 to 1.27), which suggested the presence of publication bias [16,17].

**Figure 5:**
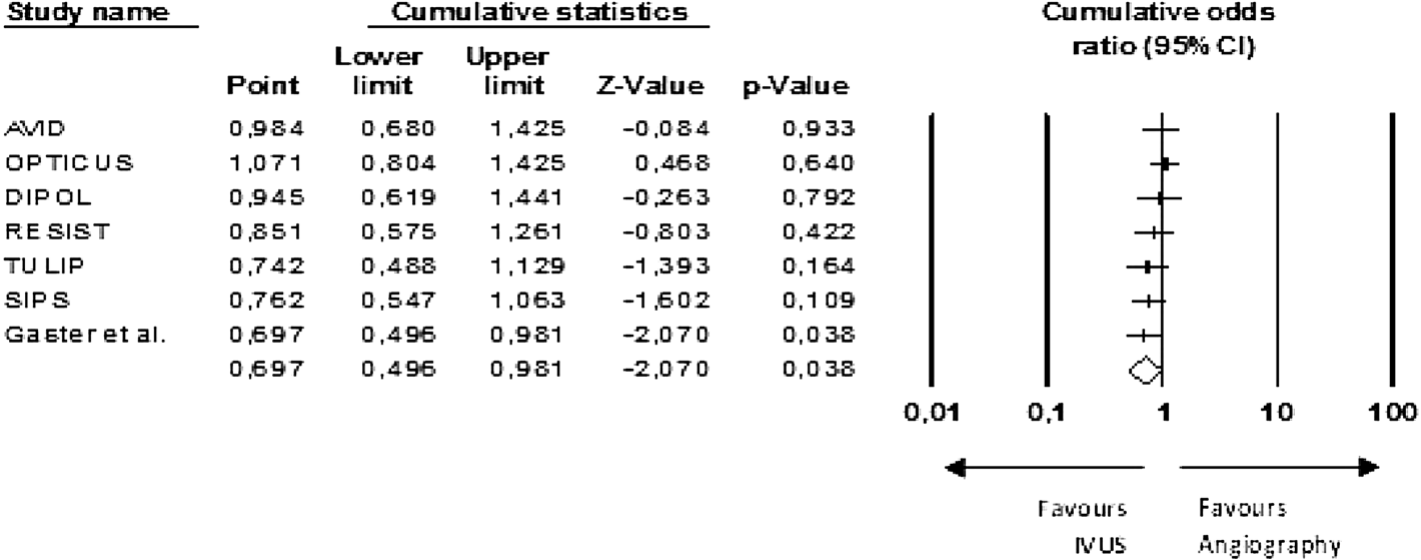
**Cumulative meta-analysis.** Each line includes the combined analysis of the corresponding study and the other ones above. The studies were added from largest to smallest sample size.

**Figure 6:**
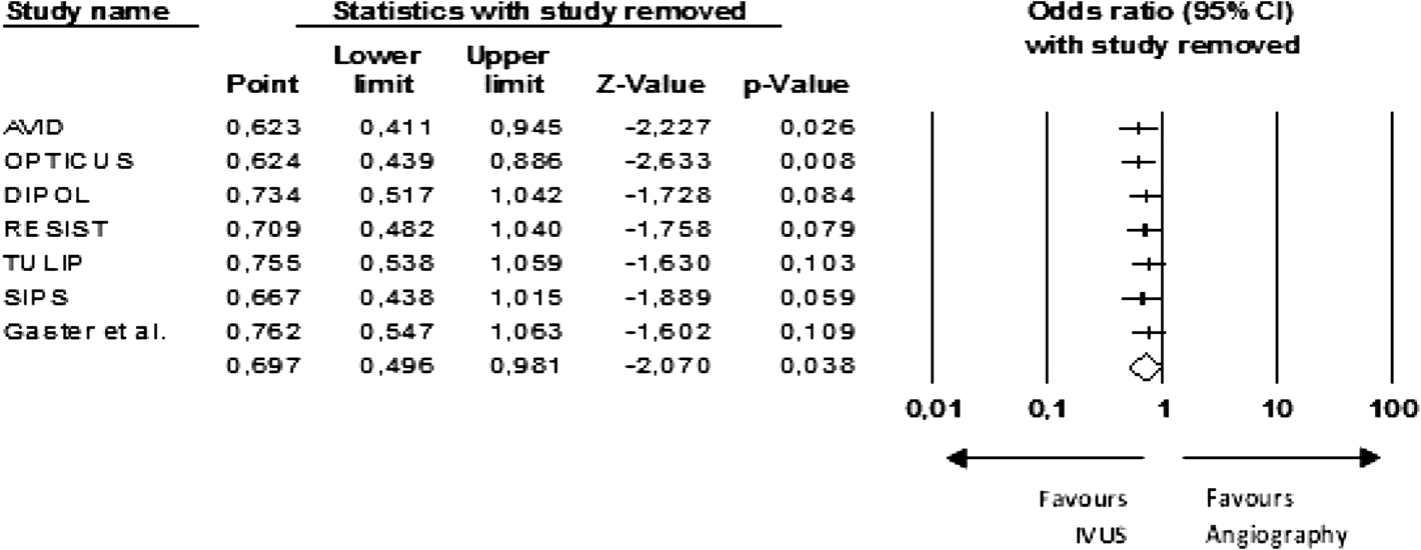
**One-study-removed method.** Each line excludes the corresponding study from the combined analysis.

A cumulative meta-analysis by reverse order of sample size was performed. The results only became positive when the last and smallest study was included in the analysis (Figure 5). Moreover, the one-study-removed analysis showed that the removal of any one of the smaller studies gave a neutral result from the meta-analysis (Figure 6). This makes it plausible to assume that one small unpublished study with negative results would be enough to nullify the effect of that meta-analysis.

## Discussion

In this rigorously conducted meta-analysis of randomized controlled trials that compared IVUS-guided PCI with angiography-guided PCI using bare metal stents, we did not find any advantage of the IVUS-guided strategy over the standard method in clinically relevant outcomes. Indeed, we found evidence of publication bias and of significant heterogeneity among the studies regarding the outcomes MI and MACE. These results diverge from the last two meta-analyses on this topic [12,13], which included studies with provisional stenting, considered surrogate outcomes, and did not evaluate the presence of publication bias. However, they are in concordance with recently published studies of IVUS-guided PCI with drug-eluting stent implantation, which were not associated with significant clinical benefits [30,31].

### Differences between selected studies

The five selected studies have important differences that might lead to completely different outcomes in another context. For example, the exclusion of patients presenting with ACS may have led to a reduction in post-interventional adverse events [32-35]. In the AVID trial, pre-interventional IVUS was not performed, which excluded an important phase of the method because one of the roles of IVUS is to assess the target lesion to help in the choice of technique and devices for the PCI [8]. Only the OPTICUS study used the MUSIC study criteria for optimal stent implantation [10], which theoretically could be associated with a lower MACE rate [36]. Angiographic follow-up was performed in most studies, which may have led to an overestimated rate of repeat revascularization, due to the oculo-stenotic reflex [37], which is the predisposition to indicate a PCI for any significant luminal obstruction, despite the presence or absence of myocardial ischemia [38]. The fact that most studies have used more comprehensive criteria for repeat revascularization may have also increased MACE rates [18,20,24].

### Study biases

The data analysis suggested the presence of publication bias in both meta-analyses. This bias may have led to apparently positive results that could be easily modified by unpublished studies with small sample sizes. It may be harmful because it can maintain or amplify an apparent beneficial effect of the intervention [39].

Significance-chasing bias is an enticing term that refers to the clustering of the most common types of meta-analysis bias, including those in which the apparently negative results remain unpublished (study publication bias and selective outcome reporting bias), those in which negative results become positive (selective analysis reporting bias) and those in which no existing data are presented as positive (fabrication bias) [40,41].

Selective reporting bias is the most common problem in meta-analyses. In selective outcome reporting bias, specific data with a negative result are omitted from publication. In selective analysis reporting bias, which is even more frequent, a negative result calculated from a pre-determined analysis plan is replaced by a positive result achieved through post hoc data analysis [41].

Another major problem is the potential presence of interests other than scientific truth. This matter becomes critical when the object of the meta-analysis is an industry product, as in the present study, because most of the researches are conducted or funded by manufacturers (Table 4) [42,43]. There is a current trend towards opposing this practice [44].

**Table 4:** Study funding and conflicts of interest of the authors of original articles included in this meta-analysis

**Trial**	**Industry funding**	**Conflicts of interest (authors)**
**DIPOL**	None declared	N/A
**AVID**	None declared	Accumetrics; Baxter; BDS; Boston Scientific; Cardium; Conor Medical; Cordis; Johnson & Johnson; Medtronic; Volcano
**RESIST**	None declared	N/A
**TULIP**	Medtronic; AVE	Boston Scientific; Guidant
**OPTICUS**	Boston Scientific; Johnson & Johnson	N/A

N/A, not applicable.

Meta-analyses have gained prestige over time but they are still considered by some to be an ancillary method, accepted only when it corroborates the point of view of experts and of public policies [41]. They could play a fundamental role in changing (or in supporting) the evidence on relevant issues if conducted properly, with a pre-specified analysis plan and declared conflicts of interest for every study included, in addition to determining and reporting all possible biases.

### Limitations

The paucity of randomized controlled trials comparing IVUS-guided PCI and angio-guided PCI and the exclusion of groups with specific lesions (long lesions, small vessels, bifurcations or left main coronary artery) may have masked a possible benefit. The same point applies to the exclusion of patients presenting with ACS, whose rate of cardiovascular events is higher, and PCI, when indicated, may even reduce mortality [32-34].

The low statistical power of the present study and of the re-evaluated meta-analysis [13] is due to the presence of heterogeneity and to the possibility of study publication bias. The presence of other biases might be possible but that is even more difficult to prove.

## Conclusion

The clinical benefit of IVUS-guided PCI with bare-metal stent implantation could be determined neither by the meta-analysis presented in this study nor by the re-evaluated meta-analysis. This may be explained by the paucity and heterogeneity of the studies published so far. Furthermore, both meta-analyses showed possible publication biases.

Therefore, there is no evidence so far to recommend routine IVUS-guided PCI with bare-metal stent implantation. Studies on specific subgroups and performance of a simple large randomized trial could show different results.

This research was conducted by public funding from the National Council for Scientific and Technological Development (CNPq) (CNPq Project: 559584/2009-1).

## Competing interest

The authors declare no conflicts of interest in the preparation and in the presentation of this meta-analysis.

## Authors’ contributions

LLJ participated in the design of the study, retrieved, reviewed and selected the articles from electronic and manual searches, helped to discuss the results and to draft the manuscript. MRS participated in the design of the study, performed the statistical analysis and helped to discuss the results and to draft the manuscript. LCP retrieved, reviewed and selected the articles from electronic and manual searches. SMBK participated in the design of the study and helped to retrieve articles from electronic searches, to discuss the results and to draft the manuscript. CFSA participated in the design of the study and helped to discuss the results and to draft the manuscript. ALR participated in the coordination of the study, helped to select the articles, to discuss the results and to draft the manuscript. All authors read and approved the final manuscript.
